# Wide prevalence of hybridization in two sympatric grasshopper species may be shaped by their relative abundances

**DOI:** 10.1186/s12862-015-0460-8

**Published:** 2015-09-16

**Authors:** Katja Rohde, Yvonne Hau, Jessica Weyer, Axel Hochkirch

**Affiliations:** Department of Biogeography, Trier University, D-54286 Trier, Germany

## Abstract

**Background:**

Hybridization between species is of conservation concern as it might threaten the genetic integrity of species. Anthropogenic factors can alter hybridization dynamics by introducing new potentially hybridizing species or by diminishing barriers to hybridization. This may even affect sympatric species pairs through environmental change, which so far has received little attention. We studied hybridization prevalence and the underlying behavioral mechanisms in two sympatric grasshopper species, a rare specialist (*Chorthippus montanus*) and a common generalist (*Chorthippus parallelus*). We conducted a mate choice experiment with constant intraspecific density and varying heterospecific density, i.e. varying relative frequency of both species.

**Results:**

Mate choice was frequency-dependent in both species with a higher risk of cross-mating with increasing heterospecific frequency, while conspecific mating increased linearly with increasing conspecific density. This illustrates that reproductive barriers could be altered by environmental change, if the relative frequency of species pairs is affected. Moreover, we performed a microsatellite analysis to detect hybridization in twelve syntopic populations (and four allotopic populations). Hybrids were detected in nearly all syntopic populations with hybridization rates reaching up to 8.9 %. Genetic diversity increased for both species when hybrids were included in the data set, but only in the common species a positive correlation between hybridization rate and genetic diversity was detected.

**Conclusion:**

Our study illustrates that the relative frequency of the two species strongly determines the effectiveness of reproductive barriers and that even the more choosy species (*Ch. montanus*) may face a higher risk of hybridization if population size decreases and its relative frequency becomes low compared to its sister species. The asymmetric mate preferences of both species may lead to quasi-unidirectional gene flow caused by unidirectional backcrossing. This might explain why genetic diversity increased only in the common species, but not in the rare one. Altogether, the hybridization rate was much higher than expected for a widely sympatric species pair.

**Electronic supplementary material:**

The online version of this article (doi:10.1186/s12862-015-0460-8) contains supplementary material, which is available to authorized users.

## Background

The impact of hybridization and the underlying mechanisms have become fascinating fields of research for evolutionary biologists and conservation biologists [1–3]. The causes and consequences vary among species. While natural hybridization is recognized as a significant evolutionary process [4, 5], anthropogenic hybridization is often negatively valued by conservation biologists [1]. However, the potential outcomes of hybridization probably do not differ between natural and anthropogenic scenarios. Hybridization can trigger speciation and could lead to new adaptations in a changing environment [4, 6–8]. It can increase genetic diversity if hybrids are fertile, niches are available and both parental species have a high fitness [9]. Furthermore, hybridization could counteract negative effects of a small population size such as inbreeding depression [3, 10] and could thus protect a species against extinction [11]. However, hybridization can also trigger the collapse of populations (and species) by genetic displacement [12] and thus the negative effects of hybridization on rare species dominate the discussion in conservation biology [1, 13, 14].

The main natural scenarios, in which hybridization takes place, are secondary contact zones of species after postglacial range expansions [15–17]. Anthropogenic drivers of hybridization include habitat loss, breakdown of ecological barriers or introduction of non-native or domesticated species [1, 6, 13, 18–21]. Most studies on natural hybridization focus on parapatric species in secondary contact zones, whereas hybridization between widely sympatric species received only little attention [2, 15, 22–24]. Even though there is a recent increase in studies on speciation with gene-flow (e.g. [25]), it is often assumed that sympatric species have evolved reproductive barriers that allow them to coexist [2, 26]. However, sympatric species do not necessarily occur in syntopy, i.e. they might differ in habitat affiliation, and thus might show a micro-allopatric distribution with several local hybrid zones (mosaic hybrid zones). Allotopy can reduce the negative effects of hybridization, but also might evolve as a consequence of such negative effects [27]. Even natural hybrid zones are influenced by anthropogenic factors and may for example be moving as a response of local hybridization equilibria to global warming [28]. Similar changes might occur for species pairs with allotopic distribution patterns, e.g. if ecological barriers break down due to habitat deterioration or alteration [24, 29, 30]. It is thus of high interest to study the patterns of hybridization in species pairs which are widely sympatric but only locally syntopic.

We investigated the hybridization prevalence and the underlying behavioral mechanisms in two sympatric grasshopper species, a rare specialist (*Chorthippus montanus,* Charpentier, 1825) and a common generalist (*Chorthippus parallelus,* Zetterstedt, 1821), which occur sympatrically in large parts of Eurasia. While *Ch. montanus* is a habitat specialist occurring in permanently moist habitats, *Ch. parallelus* is a habitat generalist which occurs in a variety of grassland habitats [31]. *Ch. parallelus* is well known as a model species for hybridization studies, forming one of the best studied hybrid zones with an Iberian subspecies in the Pyrenees [17, 32]. Previous studies have even shown that *Ch. parallelus* and *Ch. montanus* hybridize under laboratory conditions and that hybrids are fertile at least to the F2-generation [2, 33, 34]. Juvenile mortality of *Ch. montanus♂* - *Ch. parallelus*♀ hybrids is 34 % higher than in the parental species, while in *Ch. parallelus♂* - *Ch. montanus*♀ hybrids it is even lower than in the parental species. Egg mortality is 10 % lower in F1 hybrids and 16 % lower in F2 hybrids than in the parental species [34]. Both species are closely related and morphologically very similar, but differ in ecology [2, 31, 35]. Their songs have a similar structure, but differ in speed (*Ch. montanus* sings slower) [2, 33, 35]. Due to their close relationship and similar songs, and based upon the occurrence of intermediate phenotypes, hybridization has been suggested to occur in syntopic populations [35]. *Ch. montanus* is threatened by drainage of wetlands, abandonment of meadows, habitat fragmentation and increasing length of droughts [36]. During the last decades it has disappeared from nearly all sites <400 m asl in our study region, suggesting that it may be strongly affected by climate change. It is thus of high interest to explore, whether hybridization might act as an additional threat for *Ch. montanus* and if it may increase in declining populations.

Hochkirch and Lemke [2] demonstrated that females of *Ch. montanus* strongly prefer conspecific males as mates, whereas such a preference was not found for females of *Ch. parallelus* or males of both species. This may present at least a unidirectional pre-mating barrier which may reduce the hybridization probability between both species. However, it is well known that encounter rate is a major factor influencing mate choice and choosiness of females [37–39] and that previous exposure to heterospecifics may increase hybridization risk [40]. Thus, we assumed that the encounter probability of heterospecific males strongly influences female mate choice also in *Ch. montanus* and that high heterospecific frequencies (i.e. skewed abundances) may trigger interspecific matings also between *Ch. montanus* females and *Ch. parallelus* males. We further hypothesized that the ongoing decline of *Ch. montanus* may increase heterospecific encounter probabilities and thus hybridization risk to increase with decreasing population size. In order to test this hypothesis, we first performed a mate choice experiment, in which we analyzed the role of heterospecific density on mate choice when conspecific density remains constant. We expected an increasing hybridization risk with increasing heterospecific frequency and a linear increase of conspecific matings with increasing conspecific frequency. As hybridization was only proven under laboratory conditions it also aimed to test for the prevalence and extent of hybridization in the field. Therefore, we performed a microsatellite analysis in twelve syntopic and four allotopic populations. In order to detect potential drivers of hybridization and to test the hypothesis that hybridization risk increases with decreasing population size, we analyzed the hybridization rates for correlations with effective population size. As there is a strong altitudinal pattern in the decline of *Ch. montanus*, we also tested for correlations of hybridization rate and altitude. Finally, we examined the impact of hybridization on the genetic diversity of both species [12] in order to assess the direction of gene flow and to test for differences between the habitat specialist and the generalist.

## Methods

### Study species

**Fig. 1:**
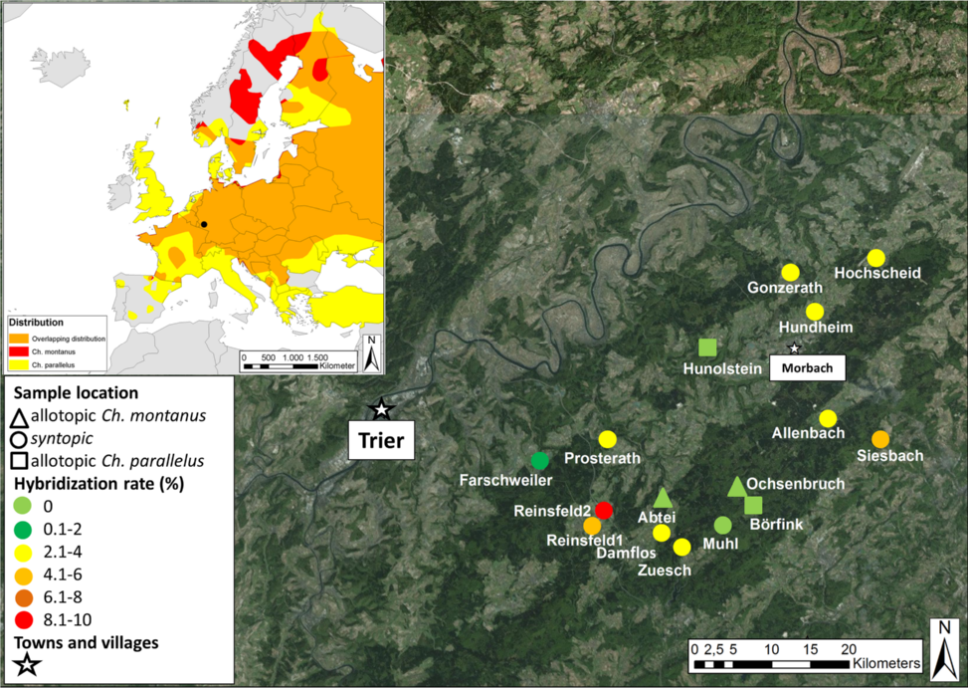
Top left: Distribution of *Ch. montanus* (*red*), *Ch. parallelus* (*yellow*) and the overlapping distribution of both species (*orange*) (distributional data from Kleukers [43]). Geographic map of each sample location in the Hunsrueck mountains and hybridization rate at each sample location (for geographic coordinates see Table 2). Triangle: allotopic population of *Ch. montanus*; square: allotopic population of *Ch. parallelus*; circle: syntopic populations

*Chorthippus montanus* is a univoltine, hygrophilous grasshopper species, which occurs in moist habitat types such as marshes, peat bogs and water meadows [41–43]. The species is listed as threatened on red lists of several European countries [36]. In the study area (Fig. 1), it has a highly fragmented distribution and is mainly found on isolated wet meadows at altitudes >400 m asl. On most of these meadows *Ch. parallelus* occurs, too, but the latter species usually occupies drier areas surrounding the wet habitat of *Ch. montanus*. Nymphs of *Ch. parallelus* hatch earlier than those of *Ch. montanus* and become adult ca. one month earlier [2, 44]. Adults of both species co-occur at least over a period of two to three months. Both species are flightless, but occasionally macropterous individuals occur, which are believed to represent the main dispersal units [31, 43]. Hybrids of both species produce intermediate songs and are morphologically either intermediate or similar to *Ch. parallelus* [2, 34].

The collection of genetic samples and live specimen for this research was permitted by the “Struktur- and Genehmigungsdirektion Nord” Rhineland-Palatinate.

### Mate choice experiment

Nymphs of *Ch. parallelus* were collected on 30 June, those of *Ch. montanus* on 06 August 2010 at three meadows: Prosterath (49°44′6.59"N; 06°54′12.87"E), Damflos (49°40′4.18"N, 06°59′33.52"E) and Hoxel (49°46′22.16"N; 07°06′9.44"E). Nymphs were reared in plastic terraria (30 × 19.5 × 20.5 cm) covered with soil and planted with grass, kept in climate chambers (Kälte Kamrath) at 25 °C and 65 % RF. They were watered each day. Aeration was ensured with a mesh lid. Each terrarium was illuminated by two UV- and VIS emitting fluorescent tubes (Osram Biolux® L36W/965). Nymphs were raised in single species groups. Adult individuals were sorted out daily by species (based upon their morphology) and sex to ensure virginity (grasshoppers become sexually mature 1–2 weeks after final moult) and to ensure that females had no previous experience with any potential mates. Mate choice experiments took place in similar terraria with moist soil and grasses. We used a full factorial design with 40 replicates of four different factor levels (frequencies) for both species (Table 1). During each replicate we observed mate choice for 90 min at four different frequencies with one pair of the target species and either one, two, three or four heterospecific pairs (non-target pairs) (1:1, 1:2, 1:3, 1:4; Table 1). At each day, we conducted 3–9 replicates with randomly chosen factor levels. The terraria were inspected every 5 min (copulations last on average 37 min ranging from 15 to 90 min [45]) and all copulations were noted (time; type of copulation: target species conspecific, target female with heterospecific male, target male with heterospecific female, non-target species conspecific). Whenever a copulation occurred, the individuals involved were marked with a permanent non-toxic paint marker (Edding 780) and released in the terraria again to keep the density constant. After 90 min, we sorted unmated individuals by species and sex. These individuals were never used as target species again, but males were used as non-target species in other replicates to increase the frequency of heterospecifics. Mated individuals were kept in separate terraria to breed them for later experiments.

**Table 1:** Composition of the mate choice experiment

Target species	Frequency	*Ch. montanus*		*Ch. parallelus*	
		♀	♂	♀	♂
*Ch. montanus*	1:1	1	1	1	1
	1:2	1	1	2	2
	1:3	1	1	3	3
	1:4	1	1	4	4
*Ch. parallelus*	1:1	1	1	1	1
	1:2	2	2	1	1
	1:3	3	3	1	1
	1:4	4	4	1	1

### Statistical analysis of the mate choice experiment

We analysed the effects of the explanatory variables (a) target species, (b) heterospecific density, (c) source locality and (d) time on the following response variables: (1) number of conspecific matings of the target species, (2) number of conspecific matings of the non-target species, (3) relative mating frequency of the non-target species (i.e. number of matings/pair), (4) number of interspecific matings with heterospecific males, (5) number of interspecific matings with heterospecific females, (6) time until first conspecific mating of both target and non-target species. For analysing the number of conspecific matings of the target species, we used generalized linear models (GLMs) with binomial data distribution. The number of conspecific matings of non-target species was also analysed with GLMs, but with Poisson distribution. We stepwise simplified all GLMs using the “step” function in R. As the number of interspecific matings was rather low, we analysed these data either with *χ*^2^ tests or Fisher’s exact tests (if the expected values were <5). The relative mating frequencies and the time until the first conspecific mating occurred were analysed with ANOVAs. The data were Box-Cox-transformed to infer the optimal exponent (λ) to fit the data to the models assumptions. All statistical analyses were computed in R 3.1.1 [46].

### Genetic analyses

#### Data collection

In 2009 and 2010 we sampled 1159 specimens (570 *Ch. montanus*, 561 *Ch. parallelus* and 28 intermediate morphotypes) from 16 localities in the Hunsrueck Mountains, Rhineland-Palatinate, Germany (Table 2, Fig. 1, for the exact sample size for each collected population see Table 3). We removed single hind legs of about 40 individuals per population and species. On 12 of these localities both species occurred syntopically, whereas Ochsenbruch represents a pure *Ch. montanus* population. In this case, we collected *Ch. parallelus* from a meadow in close vicinity. The localities Hunolstein and Abtei represent pure populations of *Ch. parallelus* and *Ch. montanus*, respectively, from which we only collected the respective species.

**Table 2:** Geographic coordinates of each sample location in the Hunsrueck mountains (in decimal degrees; coordinate system WGS84), abbreviations of each location and altitude (in meters)

Location	Abbreviation	X-coordinate	Y-coordinate	altitude
Siesbach	S.	7.226888	49.73729	456
Hochscheid	H.	7.217074	49.875070	507
Zuesch	Z.	7.010876	49.650941	509
Allenbach	Ab.	7.166868	49.754453	500
Muhl	M.	7.041020	49.671145	604
Hundheim	Hd.	7.152509	49.834350	473
Abtei	A.	6.966467	49.690865	500
Reinsfeld1	R1	6.883199	49.674076	480
Reinsfeld2	R2	6.899559	49.686529	525
Farschweiler	F.	6.827721	49.718864	392
Damflos	D.	6.984930	49.666523	540
Prosterath	P.	6.903598	49.735398	404
Gonzerath	G.	7.115982	49.863947	439
Ochsenbruch	O.	7.064372	49.694968	645
Börfink	B.	7.070153	49.685788	559
Hunolstein	Hust.	7.043359	49.802859	600

**Table 3:** Number of hybrids detected in each population using STRUCTURE, NewHybrids and Adegenet (conservative estimate: hybrids detected by all three programs, relaxed estimate: hybrids detected by two programs), hybridization rate (in %) and sample sizes for each population and species (pre-identified by morphology)

Pop	Sample size	Sample size	No. of hybrids conservative	No. of hybrids relaxed	Hybridization rate conservative (%)	Hybridization rate relaxed (%)
	*Ch. montanus*	*Ch. parallelus*				
Abtei	45		0	0	0	0
Allenbach	40	39	2	5	2.5	6.17
Börfink		39	0	5	0	11.63
Damflos	40	39	2	6	2.5	7.5
Farschweiler	40	39	1	5	1.3	6.25
Gonzerath	43	43	2	6	2.3	6.82
Hochscheid	47	36	2	4	2.4	4.71
Hundheim	41	39	3	7	2.4	8.54
Hunolstein		44	0	0	0	0
Muhl	40	40	0	0	0	0
Ochsenbruch	40		0	3	0	6.98
Prosterath	40	38	2	7	2.5	8.75
Reinsfeld1	36	42	5	9	6	10.84
Reinsfeld2	40	42	8	13	8.9	14.44
Siesbach	38	38	5	6	5.8	6.98
Züsch	40	39	2	3	2.5	3.7
total	570	557	34	79	3.35	8.15

#### Genotyping

DNA was extracted from hind femur muscle tissue using the DNeasy Blood & Tissue Kit (Qiagen). All individuals were genotyped at ten polymorphic microsatellite loci. Six microsatellite markers were designed for *Ch. parallelus* (BF1, BD5, BH5, BD7, BF9, CD6; Molecular Ecology Resources Primer Development Consortium et al. 2009), four were developed for *Ch. montanus* prior to this study (Additional file 1). For PCR we used the Qiagen Multiplex Mastermix in multiplexed PCR protocols for a combination of two to four loci with the following annealing temperatures (BF1, BH5, CD6, CM37: 54 °C; BD5, CM5: 48 °C; CM33, CM19: 51 °C; BD7, BF9: 58 °C). PCR tubes were filled with 10 μl reaction mixes (5.5 μl MultiplexMasterMix, 2 μl water, 1.4 μl genomic DNA (2–10 ng), 1.1 primer mix (1 μM/primer). The amplification was performed in a Multigene Gradient Thermal Cycler (Labnet) with the following PCR conditions: Initialization: 94 °C/10 min; Denaturation: 94 °C/45 s; Annealing: see primer/45 s; Extension: 72 °C/45 s; Final Extension: 72 °C/30 min; 37 cycles. Each forward primer was labeled with a fluorescent dye (FAM, HEX or TAMRA). Fragment lengths of PCR products were determined on a MEGABACE 1000 automated sequencer (GE Healthcare) and scored with Fragment Profiler 1.2 (Amersham Biosciences).

#### Simulating and detecting hybrids

**Fig. 2:**
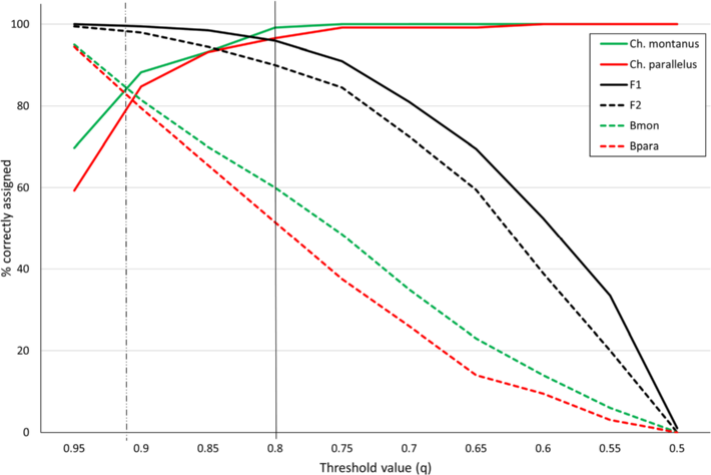
Threshold value for the correct hybrid assignment in STRUCTURE. In total 800 hybrids (200 per hybrid class, F1, F2, Backcross with *Ch. montanus* = Bmon; Backcross with *Ch. parallelus* = Bpara) were simulated with HYBRIDLAB 1.1 [51] using purebred individuals from a previous analysis (119 *Ch. montanus*, 118 *Ch. parallelus*). Afterwards the STRUCTURE run was performed with 10^5^ MCMC and a burn-in period of 10^4^ chains (with 10 iterations for *K* = 2)

In order to detect hybrids in the data set, we first simulated hybrids in HYBRIDLAB 1.1 [51]. This simulation was based upon a subset of 120 purebred individuals of each parental species, which were chosen from the complete data set after discarding potential hybrids discovered in preliminary analyses using three different programs. For the preliminary analyses we used STRUCTURE 2.3.4 and NewHybrids (both representing Bayesian approaches) and the R package adegenet 1.4–1 (which uses a discriminant analysis) [47–49]. The Structure analysis was run with the admixture model, a burn-in of 10^4^ simulations followed by 10^5^ Markov chain Monte Carlo (MCMC) simulations and a K of two with ten iterations. The posterior probability (q) belonging to one of the two clusters was used to identify hybrids without differentiation between different hybrid classes. The threshold q-value for hybrids was chosen between 0.2 and 0.8, as the simulation showed that a broader range led to an overestimate of hybridization caused by a higher number of mis-assigned pure-bred individuals, F1 and F2 hybrids (Fig. 2; Additional file 2). Hence, the threshold used here represents a conservative estimate of hybridization as has also been shown in other studies [12]. NewHybrids was developed to detect hybrids and distinguish different hybrid classes (i.e. F1, F2 and backcrosses [48]). The probabilities of each individual to belong to one of these hybrid classes were summed up and they were assigned to three categories based upon the maximum probability (i.e. either *Ch. parallelus*, *Ch. montanus* or hybrid). Posterior distributions were evaluated after 10^5^ iterations of the MCMC and a burn-in period of 10^4^ iterations. The third program adegenet 1.4–1 assigns genotypes to clusters based upon a discriminant analysis (DA), differentiating between hybrid classes. In this case, a prior assignment of all individuals to the classes is necessary. Therefore, individuals were assigned to a prior hybrid class, if this was suggested by both STRUCTURE and NewHybrids (only for the simulation study). The classification test assigned 90 % of the genotypes correctly [49].

We simulated four classes of hybrids (F1, F2 and backcrosses with both species) with 200 individuals of each class in HYBRIDLAB 1.1 [51]. HYBRIDLAB allows a maximum of 120 individuals or individuals of each parental species to be included. Therefore, we first excluded all individuals identified as potential hybrids by at least two of the abovementioned programs. We then first included all individuals, which were collected from allotopic populations. The rest of parental individuals were randomly chosen from the dataset of purebred parental species. After simulating the hybrid classes, they were added to the dataset of parental individuals and the three abovementioned programs were used to determine the accuracy of hybrid detection by the different programs using the same settings.

The original dataset was then analyzed again using STRUCTURE, NewHybrids and adegenet (with the abovementioned conditions). Each individual was finally assigned to one of three classes: (1) *Ch. parallelus*, (2) *Ch. montanus*, (3) hybrid (including F1, F2 and backcrosses) using two different approaches: In the conservative assignment, we only assigned individuals as hybrids when they were detected by all three programs. In the relaxed assignment, we assigned individuals as hybrids when they were identified by at least two of the three programs. These two approaches were used to calculate the hybridization rate for each population (hybridization rate = N_h_ / N * 100_;_*N* = Total sample size of *Ch. montanus* and *Ch. parallelus* per population, N_h_ = Number of detected hybrids). The conservative approach was used for any further analyses, whereas the relaxed approach was just calculated to obtain an upper estimate.

#### Genetic diversity

Expected and observed heterozygosities (H_e_ and H_o_) were calculated using GenAlEx 6.5 [52]. The mean number of alleles per locus (A) and allelic richness (A_R_) were analyzed in Fstat 2.9.3.2 [53]. These values were first calculated for each population of each species after excluding all hybrids detected by the conservative approach. In order to analyze the influence of hybrids on the genetic diversity of the populations, we performed a second analysis, in which we included the hybrids by assigning them to the parental population for which they had the highest assignment probability. In order to test for differences in genetic diversity in datasets with and without hybrids for each species, we only included populations where hybrids were detected and performed a paired *t*-test in R 3.0.2 [46]. Furthermore, allele frequencies, inbreeding coefficient (F_IS_) and tests of Hardy-Weinberg-Equilibrium (HWE) were calculated in GenAlEx 6.5 [52]. Fixation indices for genetic differentiation (F_ST_) between all populations of one species as well as between both species within syntopic and allotopic populations were also calculated in GenAlEx 6.5. Linkage disequilibria (based on 900 permutations and a nominal level of 1/100) between all pairs of loci were tested for each population of both species using Fstat 2.9.3.2 [53].

#### Correlation analyses

A linear regression analysis (lm) was performed in R 3.0.2 to analyze the relationship between the genetic parameters (A, A_R_, H_o,_ H_e_) of the populations (including hybrids) and hybridization rate. As we expected a higher hybridization probability with decreasing population size (based upon the mate choice experiment), we also calculated a linear regression between effective population size and hybridization rate. Effective population size (N_e_) was calculated for each population and species using ONeSAMP1.2 [54]. Here we used the datasets without potential hybrids (based upon the conservative approach) to avoid an artificial overestimation of the population size caused by the inclusion of hybrids. Finally, we analyzed the correlation between hybridization rate and altitude of the twelve syntopic populations (Table 2), because *Ch. montanus* went extinct at localities <400 m during the last decades.

## Results

### Mate choice experiment

**Fig. 3:**
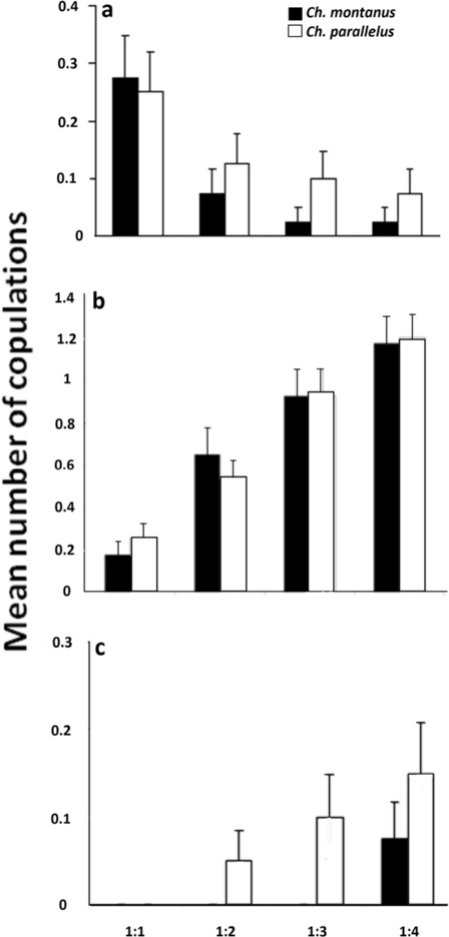
Mean number of conspecific copulations for target females of *Ch. montanus* and *Ch. parallelus* **a** with increasing heterospecific density, **b** with increasing intraspecific density, **c** Mean number of interspecific copulations for target females of *Ch. montanus* and *Ch. parallelus* with increasing heterospecific density (error bars are standard errors)

The complete number of copulations was similar among species (*Ch. montanus*: 150, *Ch. parallelus*: 155). Relative mating frequency of non-target pairs remained more or less constant among treatments (mean: 0.28 ± 0.02) and was not significantly affected by density or species. Altogether, we observed 34 interspecific matings (26 between *Ch. montanus* males and *Ch. parallelus* females and eight between *Ch. parallelus* males and *Ch. montanus* females). The number of conspecific matings of the target species did not differ significantly between species. However, it decreased in both species significantly with increasing density of heterospecifics (GLM, Rd = 196.4, df = 316, *z* = −4.02, *p* < 0,001; Fig. 3a).

The number of conspecific matings of the non-target species was also similar between species, but for both species the number of matings increased with increasing number of conspecifics (GLM, Rd = 250.9, df = 318, *z* = 7.41, *p* < 0,001; Fig. 3b). Target females of *Ch. parallelus* were more often involved in interspecific matings (12 x) than those of *Ch. montanus* (3 x; *χ*^2^ test, df = 1, *χ*^2^ = 4.48, *p* = 0.034; Fig. 3c), whereas the opposite was true for males (14 x for *Ch. montanus* males, 3 x for *Ch. parallelus* males; *χ*^2^ test, df = 1, *χ*^2^ = 6.21, *p* = 0.013). Interspecific matings of *Ch. montanus* target females were not significantly affected by density (Fisher’s Exact Test, *p* = 0.059), but only occurred at a density of 1:4, whereas in *Ch. parallelus* females the number of interspecific matings increased significantly with increasing heterospecific density (Fisher’s Exact Test, *p* = 0.045; Fig. 3c). For males, no significant effects of heterospecific density on interspecific mating frequency were found (Fisher’s Exact Test, *Ch. montanus*: *p* = 0.47, *Ch. parallelus*: *p* = 0.99). The time until a mating occurred varied between 28 and 65 minutes and was not significantly influenced by either density or species (ANOVA, *Ch. montanus*: λ = 0.64, F_1,71_ = 0.67, *p* = 0.42; *Ch. parallelus*: λ = 0.5, F_1,69_ = 0.84, *p* = 0.36).

### Hybridization rate

**Fig. 4:**
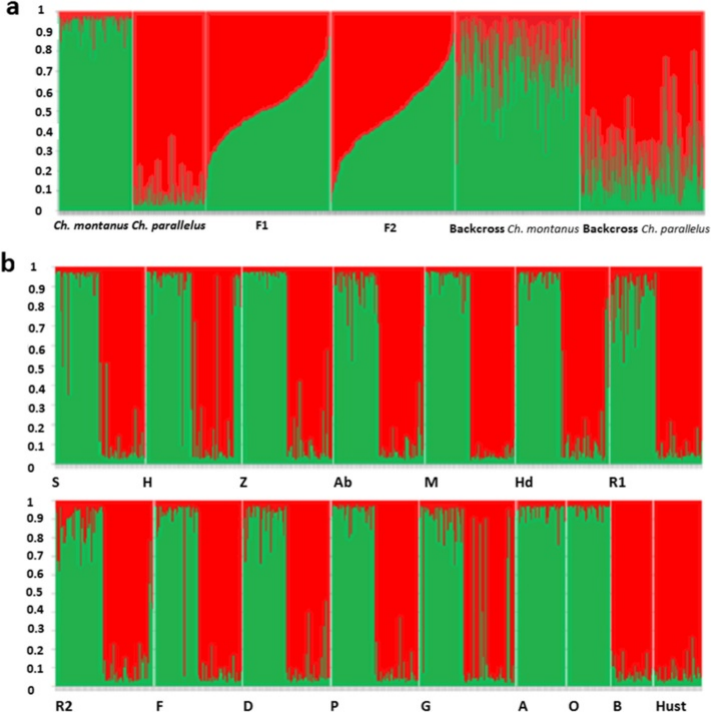
Genetic clusters found by STRUCTURE for **a** the simulated hybrid-classes and **b** the 16 sampled populations. Each individual is represented by a single vertical line, divided into K colours (*K* = 2; *Ch. montanus*: green; *Ch. parallelus*: red); the coloured segment shows the individual’s estimated proportion of membership to that genetic cluster; abbreviations correspond to **a** the simulated hybrid-classes and to **b** the 16 sampled populations. The STRUCTURE run was performed with 10^5^ MCMC and a burn-in period of 10^4^ chains (with 10 iterations for each K). Populations O and A. were allotopic populations of *Ch. montanus*, B and Hust. were allotopic populations of *Ch. parallelus*

After simulating a total of 800 hybrids (F1, F2, backcrosses with *Ch. montanus* and *Ch. parallelus*) in HYBRIDLAB, we tested the performance of the three programs by evaluating their assignment of the simulated hybrids (Additional file 2). The program NewHybrids had the best performance with an accuracy of 90 %, when hybrids were assigned to the respective hybrid class at an estimated posterior probability >0.5. Adegenet detected 88 % of the simulated hybrids correctly and STRUCTURE detected 82 % at a q value between 0.2 and 0.8 (Fig. 4a).

When we performed the same analysis with the original dataset (excluding simulated hybrids), we detected 34 hybrids using the conservative approach. With the relaxed approach we identified 79 hybrids, i.e. 46 hybrids were detected by only two programs, 23 of which were assigned as backcrosses with one of the parental species by NewHybrids and adegenet. In STRUCTURE, we assigned these individuals as purebred species at the chosen threshold of q >0.8 (Fig. 4b, Additional file 3).

The hybridization rate of all tested populations varied between 0 and 8.9 % for the conservative approach and between 0 and 14.4 % for the relaxed approach (Table 3). The highest hybridization rates were found in the populations Reinsfeld1 (conservative: 6.0; relaxed: 10.84) and Reinsfeld2 (conservative: 8.9; relaxed: 14.44). In the relaxed approach, eight hybrids were also detected in the allotopic populations Ochsenbruch and Börfink, suggesting that this approach provides an overestimate. These hybrids were assigned as backcrosses with *Ch. montanus* (3x) for Ochsenbruch and *Ch. parallelus* (5x) for Börfink. No hybrids were detected in the other allotopic populations (Hunolstein and Abtei) in any analysis.

### Genetic variability and diversity

The mean number of alleles was 11.44 ± 0.44 for *Ch. montanus* (excluding hybrids of the conservative assignment). Including the hybrids increased the mean number of alleles significantly by 8.3 % (paired *t*-test: *t* = −3.9, df = 10, *P* = 0.003; Table 4). Similarly, the number of alleles in *Ch. parallelus* populations increased from 15.6 ± 0.63 by 5.3 % when hybrids were included (paired *t*-test: *t* = −4.68, df = 10, *P* = 0.001; Table 5). When including hybrids, expected heterozygosity (H_e_) declined significantly by 1.04 % for *Ch. parallelus* (paired *t*-test: *t* = 3.89, df = 10, *P* = 0.003), but increased (not significantly) by 1.2 % for *Ch. montanus* paired *t*-test: *t* = −2.09, df = 10, *P* = 0.064; Tables 4, 5). There was no significant difference in observed heterozygosities (H_o_) between the datasets with and without hybrids (Tables 4, 5).

**Table 4:** Genetic parameters of each *Ch. montanus* population with hybrids (+) and excluding hybrids detected with the conservative approach (−)*;* N: sample size; A: mean number of alleles; H_*o*_ and H_*e*_, observed and expected heterozygosities; N*e*: mean effective population size estimate numbers in parentheses are standard errors

Pop	N +	N -	A +	A -	Ho +	Ho -	He +	He -	Ne -
S.	41	37	9.9 (1.4)	6.7 (1.1)	0.57 (0.09)	0.59 (0.09)	0.69 (0.09)	0.64 (0.09)	40.3
H.	47	46	13.2 (1.5)	12.9 (1.5)	0.66 (0.06)	0.67 (0.06)	0.81 (0.03)	0.81 (0.03)	667.7
Z.	41	40	12.0 (1.7)	11.0 (1.6)	0.69 (0.07)	0.70 (0.07)	0.80 (0.04)	0.80 (0.04)	142.7
Ab.	40	38	12.4 (1.6)	11.8 (1.6)	0.65 (0.06)	0.65 (0.06)	0.80 (0.04)	0.79 (0.04)	512.2
M.		38		12.2 (1.8)		0.71 (0.05)		0.80 (0.03)	389.4
Hd.	42	40	12.5 (1.6)	11.5 (1.7)	0.65 (0.06)	0.66 (0.06)	0.80 (0.04)	0.79 (0.04)	267.9
A.		44		10.7 (1.6)		0.60 (0.06)		0.78 (0.03)	122.3
R1.	39	35	11.5 (1.6)	10.7 (1.5)	0.67 (0.06)	0.67 (0.06)	0.79 (0.03)	0.78 (0.03)	128.4
R2.	46	38	11.9 (1.7)	11.1 (1.6)	0.57 (0.07)	0.59 (0.08)	0.75 (0.06)	0.75 (0.05)	170.9
F.	40	39	11.8 (1.7)	11.1 (1.6)	0.63 (0.06)	0.63 (0.06)	0.75 (0.04)	0.75 (0.05)	184.1
D.	39	37	13.9 (2.0)	13.3 (2.0)	0.63 (0.06)	0.63 (0.07)	0.79 (0.04)	0.79 (0.04)	656.5
P.	38	36	12.2 (1.7)	11.0 (1.6)	0.60 (0.07)	0.59 (0.07)	0.78 (0.05)	0.77 (0.05)	295.9
G.	44	42	14.7 (1.9)	14.5 (1.8)	0.68 (0.06)	0.69 (0.07)	0.80 (0.04)	0.80 (0.04)	671.01
O.		37		11.6 (1.6)		0.57 (0.07)		0.75 (0.04)	615.9
Mean	41	39	12.2 (0.4)	11.4 (0.4)	0.64 (0.02)	0.64 (0.02)	0.78 (0.01)	0.77 (0.01)

**Table 5:** Genetic parameters of each *Ch. parallelus* population with hybrids (+) and excluding hybrids detected with the conservative approach (−)*;* N: sample size; A: mean number of alleles; H_*o*_ and H_*e*_, observed and expected heterozygosities; N_*e*_: mean effective population size estimate; numbers in parentheses are standard errors

Pop.	N +	N -	A +	A -	Ho +	Ho -	He +	He -	Ne -
S.	39	34	16.7 (2.4)	15.0 (2.2)	0.58 (0.06)	0.59 (0.06)	0.80 (0.04)	0.81 (0.04)	4,097.7
H.	32	31	14.3 (1.9)	14.3 (1.9)	0.45 (0.07)	0.45 (0.07)	0.76 (0.06)	0.76 (0.06)	14,598.3
Z.	38	36	17.0 (2.6)	16.6 (2.5)	0.61 (0.07)	0.61 (0.07)	0.81 (0.06)	0.81 (0.06)	23,716.16
M.		36		14.3 (2.3)		0.59 (0.05)		0.8 (0.04)	8,574.6
Hd.	40	38	17.6 (2.6)	17.0 (2.6)	0.59 (0.04)	0.59 (0.04)	0.81 (0.04)	0.81 (0.04)	11,870.7
Ab.	32	30	14.6 (2.7)	14.0 (2.6)	0.51 (0.08)	0.51 (0.08)	0.76 (0.04)	0.76 (0.07)	4,957.3
R1.	46	41	18.8 (2.4)	17.3 (2.5)	0.68 (0.06)	0.68 (0.07)	0.77 (0.06)	0.77 (0.06)	8,819.9
R2.	49	41	18.0 (2.2)	16.2 (2.0)	0.64 (0.07)	0.67 (0.08)	0.77 (0.06)	0.77 (0.06)	4,283.3
F.	38	37	14.8 (2.4)	14.3 (2.5)	0.52 (0.09)	0.52 (0.09)	0.78 (0.07)	0.78 (0.07)	4,192.2
D.	39	37	16.0 (2.8)	15.1 (2.8)	0.58 (0.08)	0.58 (0.08)	0.77 (0.08)	0.77 (0.07)	9,989.2
P.	39	37	16.8 (2.6)	16.1 (2.5)	0.60 (0.08)	0.60 (0.08)	0.78 (0.08)	0.79 (0.07)	7,849.2
G.	43	41	16.7 (2.6)	16.3 (2.5)	0.55 (0.06)	0.55 (0.06)	0.77 (0.06)	0.78 (0.07)	19,020.4
B.		37		15.4 (2.8)		0.59 (0.08)		0.78 (0.07)	1,489.4
Hust		39		16.5 (2.4)		0.62 (0.05)		0.82 (0.04)	10,285.4
Mean	39	37	16.3 (0.6)	15.6 (0.6)	0.58 (0.02)	0.58 (0.02)	0.80 (0.02)	0.78 (0.02)	

For some loci species-specific alleles were evident, e.g. in locus CM33 alleles 298–313 were common in *Ch. parallelus* but rare in *Ch. montanus*, while alleles 316–328 were common in *Ch. montanus* and rare in *Ch. parallelus* (Additional file 4). We found no linkage disequilibria for any locus combination (Additional file 5). Many loci deviated significant from HWE (Additional file 6). F_IS_ values were generally positive, independent of whether hybrids were included in the data set or not. Even though the number of significant deviations from HWE increased in *Ch. montanus* when including hybrids, FIS values showed no significant decrease (or increase). F_ST_ values between species were significantly lower when hybrids were included than when excluding hybrids (paired *t*-test: *t* = 2.94, df = 13, *P* = 0.012; Additional file 7). FST values between populations within one species increased significantly when excluding hybrids (*Ch. montanus*: paired *t*-test: *t* = 4.04, df = 13, *P* = 0.0014; *Ch. parallelus*: paired *t*-test: *t* = 3.84, df = 13, *P* = 0.002; Additional file 7).

### Correlation Analyses

**Fig. 5:**
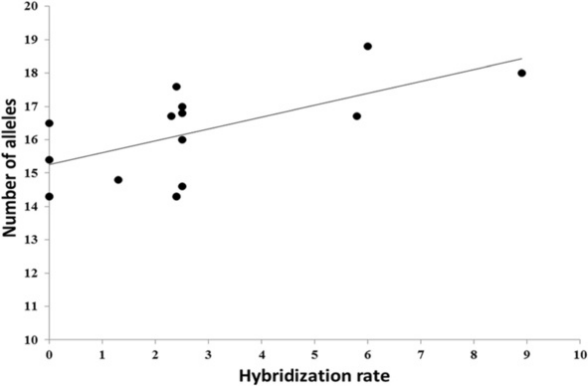
Correlation between hybridization rate (%) and number of alleles (A) of *Ch. parallelus* populations (R^2^ = 0.41)

We found no significant correlation between hybridization rate and any genetic parameter for *Ch. montanus* or altitude. However, for *Ch. parallelus* we found a significant positive correlation between hybridization rate and the number of alleles (R^2^ = 0.41, F_1,12_ = 8.3, *P* = 0.014; Fig. 5). The correlation of hybridization rate and N_e_ was not significant, but for the populations of *Ch. montanus* there was a rather high coefficient of determination (R^2^ = 0.22, F_1,10_ = 2.8, *P* = 0.126) with hybridization rate increasing with decreasing N_e_.

## Discussion

Despite the widespread assumption that hybridization between sympatric species is rare, our results show that even species with broadly overlapping ranges hybridize in nature. Although the two grasshopper species differ in their habitat requirements and phenology, niche overlap is strong enough to allow a considerable amount of heterospecific encounters in the field (twelve of the 14 *Ch. montanus* populations were in contact with *Ch. parallelus*). Nevertheless, hybridization rate seems to be low enough to prevent a complete admixture of populations of both species. Furthermore, our lab experiment shows that hybridization risk increases with decreasing population size, i.e. increasing heterospecific encounter frequency (while increasing conspecific density did not affect the individual mating frequencies for both species). *Ch. montanus* is sensitive to droughts and habitat deterioration and has shown considerable population decline in the study area (Rohde unpublished observations), whereas *Ch. parallelus* has stable (or even increasing) populations. This suggests that small *Ch. montanus* populations might face an additional risk of being genetically displaced by *Ch. parallelus*.

### Evidence of hybridization

Natural hybridization between *Ch. montanus* and *Ch. parallelus* was first proposed by Chládek [55], who found individuals with mixed morphological characters in Slovakia. However, these morphological intermediate individuals from the Slovakian Tatry Mts. have meanwhile been described as a new species, *Chorthippus smardai* [56]. Reynolds [35] also recognized morphologically intermediate individuals and suspected hybridization in the wild. Other studies have shown that these two species hybridize at least under laboratory conditions with very low fitness loss of the F1 and F2 generations [2, 34]. Our study provides the first genetic evidence that both species hybridize also in nature. In nearly all syntopic populations (except for Muhl) we identified hybrids. The hybridization rate reached a maximum of 8.9 % (but may reach up to 14.44 % when accepting the relaxed approach). The three programs varied in hybrid detection accuracy with NewHybrids performing best. Nevertheless, we recommend our approach of using all three programs as well as a prior simulation of hybrids to avoid an overestimation by a single program. With the relaxed approach we even detected hybrids in allotopic populations, which we believe to be unrealistic, even though one might argue that macropterous heterospecific individuals might occasionally immigrate. It also must be considered that STRUCTURE distinguishes neither hybrid generation nor backcrosses, which could lead to mis-assignments in some cases, leading to a more conservative estimate.

*Ch. montanus* and *Ch. parallelus* occur sympatrically in large parts of the Palearctic. It is thus surprising that both species regularly hybridize in nature. However, the contact between both species might be rather recent (in evolutionary terms), because *Ch. parallelus* recolonized large parts of its range during the postglacial period from Mediterranean refugia [57, 58]. The colonization history of *Ch. montanus* has not been reconstructed yet, but it does not occur in the Mediterranean and is generally found further north [43, 59]. This suggests that it might have colonized the temperate zone earlier or even survived here during the last glacial maximum. Hence, one may speculate that *Ch. montanus* reached its large geographic range earlier. With ongoing warming it might have become more and more restricted to higher altitudes and came in contact with *Ch. parallelus* that still expands its range [60].

As we found hybrids in nearly all populations and hybrids are known to have nearly no fitness loss [34], the question arises why the species do not mix up completely and build hybrid swarms [12]. Either the hybridization rate is still low enough to avoid complete admixture, or hybrid fitness is much lower in the field than in the lab, possibly due to mismatches of traits acting as premating barriers. Three premating barriers are usually considered important for this species pair: (1) distinct songs of both species [61], (2) differing habitat preferences, resulting from specific drought sensitivity of the eggs [34, 41], (3) differences in the phenology with *Ch. parallelus* becoming adult ca. one month earlier than *Ch. montanus* [34]. It has recently been shown that the latter two aspects substantially reduce hybridization risk of both species in the field (Rohde unpublished observations). Hybrids have intermediate habitat preferences and phenologies. Thus, it is unlikely that these aspects will act as efficient barriers to backcrossing of hybrids. The intermediate song of hybrids [2] might indeed act as an efficient barrier to backcrossing hybrids, but the song differences of the parental species are much stronger and should prevent hybridization in the first place. Hence, it remains unresolved, if the lack of complete admixture is caused by such barriers or by the low hybridization rate. It is also possible that backcrosses mainly occur in one direction (with *Ch. parallelus* females), so that quasi-unidirectional gene flow occurs.

### Population size and hybridization risk

Our results confirm that females of *Ch. montanus* have a much stronger preference for conspecific males than females of *Ch. parallelus* [2]. Such an asymmetric reproductive isolation seems to be common and is attributed to the ecological and reproductive differences among sexes and species [27, 62, 63]. Differences in courtship songs of both species or even dissimilarities in pheromones (cuticular hydrocarbons) between both species could provide the underlying mechanism in the discrimination of *Ch. montanus* [64, 65]. However, the role of pheromones in mate choice of these species has not been studied so far. This unidirectional barrier combined with the differences in habitat requirements and phenology may protect natural populations of *Ch. montanus* from rapid admixture with *Ch. parallelus*. This would be in line with the assumption that multiple barriers cause restriction of gene flow between closely related species [66]. We assume that the asymmetry in female choosiness has evolved as a consequence of the different encounter probabilities caused by their differing ecology and distribution. While most *Ch. montanus* populations occur in syntopy with *Ch. parallelus*, the latter species has a very wide distribution and occurs only rarely syntopically with *Ch. montanus*. Therefore, selection pressure on reproductive barriers affects a higher proportion of *Ch. montanus* females, but only a very small proportion of *Ch. parallelus* females [62, 67]. However, it is also possible that the lower choosiness is caused by the age of females. As *Ch. parallelus* becomes adult earlier, they might have a reduced choosiness (i.e. higher receptivity) than those of *Ch. montanus*, which are still younger.

The records of hybrids from natural populations show that hybridization is not an artifact produced by laboratory conditions. It confirms that interspecific mating occurs regularly in the wild despite the existence of ecological, phenological and ethological barriers. Mate choice strongly depends on the encounter rate of potential mates and the costs and benefits of mate choice [37, 39]. Low encounter rates with conspecific mates increase the costs of mate searching and reduce choosiness [37, 39]. Our mate choice experiment demonstrates a decreasing frequency of conspecific matings and an increasing number of cross-matings with increasing heterospecific density for females of both species, but females of *Ch. montanus* only chose heterospecific males at the highest density of heterospecifics (1:4). This suggests that even the bioacoustic differences of both species are not sufficient to ensure a “correct” mate choice. If the direct contact of individuals is more important for mate finding than the song, the encounter probabilities might determine hybridization risk [27]. Songs may only be important at low densities to find corresponding mates [44, 68].

The results of our lab experiment suggest that demography might be a major driver of hybridization in wild syntopic populations. We suppose that in large populations of *Ch. montanus* hybridization is rare and restricted to the periphery of the habitat, which might lead to the formation of a mosaic hybrid zone, but not to genetic displacement [34]. If a *Ch. montanus* population decreases in size and abundances become more and more skewed towards *Ch. parallelus*, the reproductive barrier might weaken as has been shown for other rare species [39, 62, 69, 70]. Hence, a population decline caused by land use change (abandonment), drainage or climate change [6, 36] might lead to a vortex effect, increasing the strength of other threats such as hybridization. In fact, we monitored the population dynamics of the R1 and R2 populations from 2010 to 2014 (Rohde unpublished observations) and found that *Ch. montanus* declined by 90.3 % on R1 and by 49.6 % on R2 during this period. We assume that the decline was mainly driven by weather conditions (there were severe droughts in spring and autumn 2011, which might have caused the severe population decline of R1 by 87 % until 2012) or ongoing accumulation of grass debris at the sites due to abandonment. However, this population decline might increase the risk of future hybridization or even might be increasingly caused by hybridization itself.

### Genetic diversity and hybridization

It is well known and consistent that hybridization increases genetic diversity within a population [12]. Population augmentation is therefore sometimes used in conservation management to avoid inbreeding depression at low population size [71]. As long as *Ch. montanus* populations remain large and stable, a leaky reproductive isolation could increase genetic variability [3, 10, 72]. However, hybridization can also lead to a near-complete genetic displacement of a species. Hedrick [73] compared introgression of red wolf populations from coyote populations with Wright’s continent-island model [74], i.e. with unidirectional gene flow. This is probably an oversimplification as gene flow would necessarily affect both populations and thus would follow Wright’s general island model, i.e. gene flow in both directions. This means that the larger gene pool of *Ch. parallelus* will displace the gene pool of *Ch. montanus* until an equilibrium is reached. A new, completely admixed population will thus conserve some *Ch. montanus* alleles at a very low frequency (reinforced by heterosis; [75]). This is similar to Neanderthal alleles being still present in the human gene pool [76], but the genetic integrity of the *Ch. montanus* population would be lost [70]. By contrast, the genetic diversity of large populations of *Ch. parallelus* increases with occasional hybridization. It remains unknown, whether this may represent an advantage (higher adaptability) or a risk (genetic incompatibilities) in the long term.

## Conclusions

Our results support the hypothesis that hybridization between the sympatric sister species *Ch. montanus* and *Ch. parallelus* also occurs in the wild. We assume that naturally hybridization mainly takes place in ecotones between wetlands and drier habitat types, where both species come into contact. As cross-mating probability increased in the lab with decreasing relative frequency of conspecific mates, we conclude that the encounter rate is a major driver of hybridization. Population decline caused by stochastic and environmental fluctuation will thus increase the probability of hybridization as an additional threat. Habitat restoration and wetland management are therefore important tools to save this species from such vortex situations.

## Availability of Supporting Data

The microsatellite data of this paper are deposited at Dryad data repository (doi:10.5061/dryad.1dd0m).

## Additional files

Additional file 1:
**Characterization of four polymorphic microsatellite primers for** ***Ch. montanus*** **with: locus name; repeat motif; primer sequence of forward (for) and reverse (rev) primer; allele size range (bp) and fluorescence dye name (Tag).** (DOCX 17 kb) Additional file 2:
**Performance test of the three programs adegenet, NewHybrids and STRUCTURE (q-values 0.2–0.8 and 0.09–0.91).** Assignment of 238 purebred species and 800 simulated hybrids by each program and correct assignment to the different hybrid classes (F1, F2 and backcrosses with each parental species) by the programs adegenet and NewHybrids. (XLSX 173 kb) Additional file 3:
**Hybrid detection (conservative and relaxed), hybridization rate and assignment to the different hybrid classes (F1, F2, backcrosses with ach parental species) in the 16 sampled populations using the three programs adegenet, NewHybrids and STRUCTURE (q value: 0.2–0.8 and 0.09–0.91).** (XLSX 162 kb) Additional file 4:
**Allele frequencies by species and population for the ten microsatellite loci.** (XLSX 136 kb) Additional file 5:
**Linkage disequilibrium based on 900 permutation and a nominal level of 1/100 between each locus for each population of both species.** (XLSX 18 kb) Additional file 6:
**Deviations from Hardy-Weinberg-Equilibrium and inbreeding coefficient for both species and each population and locus.** (XLSX 50 kb) Additional file 7:
**Fixation indices for genetic differentiation (F**_**ST**_ **) between the populations within one species and between the species in syntopic and allotopic populations.** (XLSX 19 kb)
