# Supplementary material for: Wide prevalence of hybridization in two sympatric grasshopper species may be shaped by their relative abundances
Source: BMC Evol Biol. 2015 Sep 16;15:191. doi: 10.1186/s12862-015-0460-8 (PMC4573947; doi:10.1186/s12862-015-0460-8)
Supplement: Additional file 1: — Characterization of four polymorphic microsatellite primers for Ch. montanus with: locus name; repeat motif; primer sequence of forward (for) and reverse (rev) primer; allele size range (bp) and fluorescence dye name (Tag). (DOCX 17 kb) [file 12862_2015_460_MOESM1_ESM.docx]

Additional file 1: Characterization of four polymorphic microsatellite primers for *Ch. montanus* with: locus name; repeat motif; primer sequence of forward (for) and reverse (rev) primer; allele size range (bp) and fluorescence dye name (Tag).

| Locus | Repeat motif | Primer-Sequenz 5´-3´ | Allele size range (bp) | Tag |
| --- | --- | --- | --- | --- |
| CM 5 | (ATC)21 | for: TGTACCCATGAGCTACTGTCA  rev: TGGCAAACTGGCGAGCTTCT | 306-432 | HEX |
| CM 19 | (TCTG)4(TCCG)3 | for: CGATCGCCTTTTGACAGCTC  rev: CCATATTCTCGCGTGGCTTG | 410-450 | FAM |
| CM 33 | (GAT)11 | for: ACAAACTGTCTCGAATACTTGC  rev: GGTAGTAGCTATTCTTGAGTTG | 301-349 | TAMRA |
| CM 37 | (TCA)6 | for: GTTTCCGTGATCCTGAGCG  rev: AGGTACTTGGATTCGGTGAG | 219-339 | TAMRA |
